# Intra-colony light gradients drive variation in coral symbiont morphology and carbon storage

**DOI:** 10.1093/ismejo/wrag006

**Published:** 2026-01-28

**Authors:** Andrea Catacora-Grundy, Netanel Kramer, Sofie Lindegaard Jakobsen, Michael Kühl, Johan Decelle, Daniel Wangpraseurt

**Affiliations:** Cell and Plant Physiology Laboratory, CNRS, CEA, INRAE, IRIG, University Grenoble Alpes, 17 avenue des Martyrs, 38054 Grenoble, France; Marine Biology Research Division, Scripps Institution of Oceanography, University of California San Diego, 9500 Gilman Dr #0202, La Jolla, CA 92093-0202, United States; Marine Biology Section, Department of Biology, University of Copenhagen, Strandpromenaden 5, 3000 Helsingør, Denmark; Marine Biology Section, Department of Biology, University of Copenhagen, Strandpromenaden 5, 3000 Helsingør, Denmark; Cell and Plant Physiology Laboratory, CNRS, CEA, INRAE, IRIG, University Grenoble Alpes, 17 avenue des Martyrs, 38054 Grenoble, France; Marine Biology Research Division, Scripps Institution of Oceanography, University of California San Diego, 9500 Gilman Dr #0202, La Jolla, CA 92093-0202, United States

**Keywords:** coral photobiology, Symbiodiniaceae, FIB-SEM, carbon storage, morphometrics, light microenvironment

## Abstract

Light availability plays a central role in shaping the photophysiology and energy metabolism of photosymbiotic organisms such as reef-building corals. Although light varies greatly within coral colonies, the effects of this spatial heterogeneity on the subcellular organization and energy storage of symbiotic algae (Symbiodiniaceae) remain poorly understood. Here, we combined microscale measurements of light and oxygen across both light-exposed upper regions and shaded basal regions of a *Favites abdita* colony with three-dimensional cellular imaging using focused ion beam scanning electron microscopy. Our multiscale approach revealed subcellular heterogeneity among symbiont populations, suggesting different cell-cycle stages and physiological states across a spatial stratification in the coral. Subcellular morphometrics revealed that symbiont cells at the top of the colony were twice as voluminous as those at the shaded base, despite similar plastid volume occupancy. Compared to symbionts at the top of the colony, symbionts in the basal region accumulated nearly three times more starch relative to their cell volume. These findings show that light gradients within coral colonies shape symbiont morphology and energy storage patterns, with important implications for coral stress tolerance and resilience.

Light is a pivotal parameter controlling photosynthesis and energy metabolism in multicellular organisms harboring symbiotic microalgae, such as reef-building corals [1]. These organisms host photosynthetic dinoflagellates of the family Symbiodiniaceae within their tissues, forming a partnership where symbionts supply O_2_ and photosynthetically fixed carbon to the host, supporting growth and metabolism [2, 3], while the coral provides shelter, inorganic carbon, and nutrients [1]. Despite extensive research on coral-symbiont biology, the influence of intra-colony light gradients on symbiont subcellular architecture and carbon dynamics remains poorly understood [4]. Symbiont populations are often treated as homogenous entities, despite dynamic light and chemical gradients within tissues that may structure the distribution and intracellular architecture of Symbiodiniaceae *in hospite* [5, 6].

Light distribution in coral tissues varies across spatial scales, from colony-wide differences to microgradients within tissue [7–9]. Incident light can differ by up to 95% between exposed and shaded regions of branching corals, and only ~10% of surface irradiance penetrates deeper tissue layers [7, 8]. Significant intra-colony variability in photosynthesis, carbon fixation, and photochemical efficiency has been documented [6, 10, 11], but subcellular responses of algal symbionts to light gradients, including changes in cell architecture, organelle size, and carbon storage, are largely unexplored [12]. To address this gap, we investigated the subcellular architecture of Symbiodiniaceae across light microhabitats within a massive *Favites abdita* colony at Heron Island, Southern Great Barrier Reef. Fragments were collected during midday sun exposure (Fig. S1) from the directly light-exposed top and shaded base of the colony (Fig. 1a; Fig. S2; Supplementary Information), corresponding to an approximately six-fold difference in scalar irradiance [7]. Using focused-ion beam scanning electron microscopy (FIB-SEM) and high-resolution microsensing, we analyzed changes in subcellular morphometrics, physiology, and the light microenvironment of Symbiodiniaceae. Microscale light and O_2_ concentration gradients were characterized with fiber-optic scalar irradiance and electrochemical O_2_ microsensors (~50 μm tip diameter) [8]. Coral tissues were sampled *in situ*, fixed, and prepared for FIB-SEM imaging ([13]; Fig. S3; Supplementary Information). A 3D reconstruction pipeline quantified subcellular components, including cell volume, plastids, and starch granules [13]. We limited the reconstruction to chloroplast and starch compartments due to imaging constraints inherent to coral tissues and the labor-intensive nature of FIB-SEM. Consistent segmentation protocols across samples ensure reliable relative quantification, despite challenges in estimating absolute volume uncertainty from contrast variability between cellular compartments. These multiscale approaches enabled us to quantitatively link light microhabitats to the subcellular architecture and energy storage of the Symbiodiniaceae at different locations in the colony. Accordingly, analyses were conducted on a limited number of cells (*n* = 9–22) due to contrast constraints and the time-intensive nature of FIB-SEM imaging, especially in coral tissues where chemical fixation and sample preparation pose additional challenges. As a first application, our aim is to demonstrate the method’s ability to detect regional differences in subcellular architecture within a single colony. We observed statistically significant differences in symbiont, starch, and chloroplast volumes between regions (permutational *t*-tests), underscoring the method’s potential to reveal colony-scale patterns.

**Figure 1:**
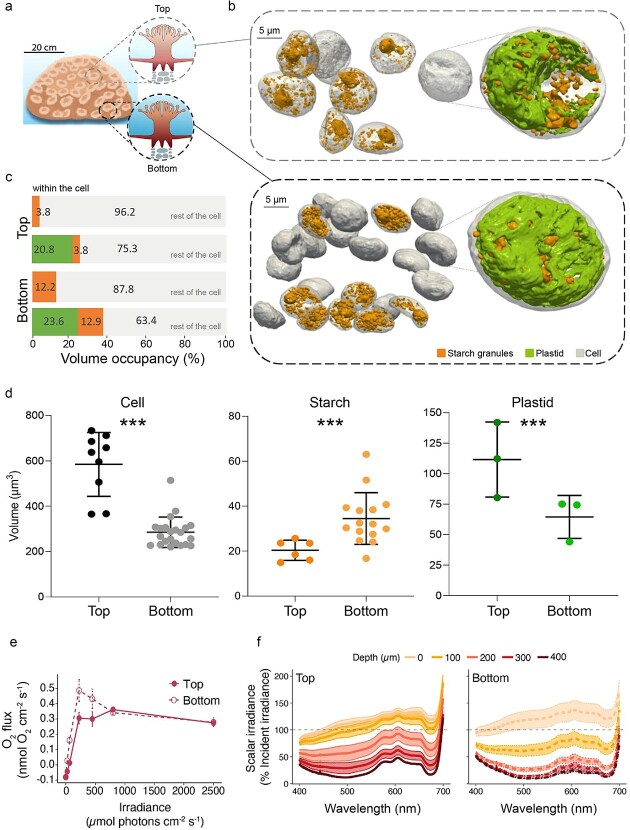
Spatial variation in light microenvironment, photosynthesis, and symbiont subcellular architecture between light-exposed and shaded regions of a *Favites abdita* colony. (a) Sketch of the massive coral *F. abdita*, illustrating the top (light-exposed) and bottom (shaded) regions of the colony selected for sampling. (b) 3D FIB-SEM reconstruction showing the intracellular organization of Symbiodiniaceae within the endodermal tissue of *F. abdita*. (c) Volume occupancy of starch granules and plastids (expressed as a percentage of total cell volume) in symbionts from the top and bottom regions of the colony. (d) Morphometric comparison of cell, starch granule, and plastids volumes (μm^3^) between the two regions, presented as mean ± SD. Asterisks (***) denote statistically significant differences (*P* = .001; permutational *t*-test). (e) Net O_2_ flux between coral coenosarc tissue and the overlaying water measured using O_2_ microsensors as a function of incident photon irradiance (data points represent means ± SD; *n* = 3–5 randomly chosen coenosarc tissue areas in the exposed and shaded parts of the coral colony, respectively). (f) Vertical microprofiles of spectral scalar irradiance in coenosarc tissue (solid lines represent mean spectra with SE is shown as colored areas; *n* = 5 random measurements in chosen tissue areas in the exposed and shaded parts of the coral colony, respectively). Scalar irradiances are normalized to incident downwelling spectral irradiance at the coral tissue surface.

Light microsensor measurements confirmed strong variation in internal light microclimates between colony regions (Fig. 1e and f). Light attenuation was strongest in shaded regions, consistent with their position, while high light-exposed top regions exhibited lower attenuation. These differences were reflected in net photosynthesis–irradiance curves, where bottom-region symbionts exhibited higher photosynthetic efficiency at low light, indicating low-light adaptation [14], while top-region symbionts exhibited greater maximum photosynthetic rates (Fig. 1e). Symbiont size and density also varied spatially in the host colony (Fig. 1a). While cells at the colony base were more densely packed (Fig. 1b), the cell volume of symbionts at the top was two-fold higher (584.37 ± 140.80 μm^3^; *n* = 8) compared to cells at the base (285.04 ± 67.12 μm^3^; *n* = 22; Fig. 1d). Although chloroplasts occupied a consistent relative volume (20.84% ± 2.6% at the top and 23.63% ± 6.2% in the bottom region—Supplementary Table S1) in microalgae from both regions (Fig. 1c), their absolute volume was 1.7 times larger in symbionts from the upper colony’s region (111.52 ± 30.78 μm^3^; *n* = 3) than in the lower colony’s region (64.48 ± 17.61 μm^3^; *n* = 3; Fig. 1d). This could suggest that symbionts modulate absolute plastid size in response to light availability without altering their overall cellular investment in the photosynthetic machinery.

Starch, the main form of carbon storage in Symbiodiniaceae, also varied spatially. Symbionts from the bottom region contained 1.7 times more starch granules (34.5 ± 11.5 μm^3^; *n* = 15) than those from the top (20.4 ± 4.5 μm^3^; *n* = 6). Relative to cell size, bottom-region symbionts had 3.2 times more starch (12.23 ± 4.35%; *n* = 15; Fig. 1d) than top-region symbionts (3.80 ± 0.94%; *n* = 6; Fig. 1d). Thus, FIB-SEM quantification revealed distinct patterns of carbon storage between the light-exposed and shaded regions of the coral, while chloroplast proportions remained stable. Increased starch in shaded symbionts may reflect reduced starch breakdown, enhanced low-light carbon fixation, or decreased translocation to the host. Under low light, reduced photosynthetic output promotes retention and storage of fixed carbon as starch [15], likely as a survival strategy. In contrast, under high light, surplus photosynthate is more rapidly exported or used in other metabolic pathways [16]. Additionally, the higher symbiont density at the colony base may itself influence carbon dynamics through altered host-symbiont signaling or nutrient competition, though disentangling density from light effects requires further experimental investigation.

Although low light typically induces larger chloroplasts to maximize light harvesting in microalgae like diatoms [13], the constant chloroplast occupancy (~22%) observed here suggests Symbiodiniaceae deploy a distinct photoacclimation strategy [17, 18]. Symbiodiniaceae may acclimate primarily by adjusting pigment content, photosynthetic unit size, and thylakoid organization rather than by large shifts in chloroplast volume [19, 20]. By revealing how intracellular symbionts adapt to light heterogeneity, our study highlights the need to view symbiont populations as physiologically heterogeneous, consistent with evidence of genetic and functional diversity within Symbiodiniaceae [21, 22]. Such heterogeneity may explain differential bleaching patterns within coral colonies, where shaded regions often retain symbionts longer during thermal stress [22].

The stratification of symbiont physiology documented here has important implications for coral stress responses. We speculate that the higher starch accumulation in shaded symbionts could reflect reduced carbon translocation to the host, but alternatively, larger energy reserves may render these symbionts more tolerant to bleaching. The latter interpretation aligns with field observations, where shaded regions often retain symbionts longer during thermal stress, while exposed regions bleach first. However, such an algal symbiont-centric view overlooks host metabolism and the heterogeneous distribution of holobiont-associated microbiomes across colonies [22, 23]. These intra-colony differences highlight the complex interplay between light environments, symbiont, microbiome, and host physiology that ultimately determines coral health. Future research should integrate morphometric approaches with molecular profiling to map microbial diversity and gene expression patterns across light gradients, coupled with physiological measurements of carbon fixation and translocation rates, to elucidate the molecular mechanisms underlying the observed subcellular heterogeneities in Symbiodiniaceae and their functional consequences for coral holobiont fitness.

## Supplementary Material

supplementary_materials_wrag006

## Data Availability

The datasets generated during and/or analyzed during the current study are available in the Figshare repository, https://doi.org/10.6084/m9.figshare.29944757.
